# Increased neutrophil extracellular traps activate NLRP3 and inflammatory macrophages in adult-onset Still’s disease

**DOI:** 10.1186/s13075-018-1800-z

**Published:** 2019-01-07

**Authors:** Qiongyi Hu, Hui Shi, Ting Zeng, Honglei Liu, Yutong Su, Xiaobing Cheng, Junna Ye, Yufeng Yin, Mengru Liu, Hui Zheng, Xinyao Wu, Huihui Chi, Zhuochao Zhou, Jinchao Jia, Yue Sun, Jialin Teng, Chengde Yang

**Affiliations:** Department of Rheumatology and Immunology, Ruijin Hospital, Shanghai Jiao Tong University School of Medicine, No. 197 Ruijin Second Road, Shanghai, 200025 China

**Keywords:** Adult-onset Still’s disease, Neutrophil extracellular traps, NLRP3 inflammasome, Macrophages

## Abstract

**Background:**

Adult-onset Still’s disease (AOSD) is a systemic inflammatory disease characterized by neutrophilia and NLRP3 inflammasome and macrophage activation. We investigated the role of neutrophil extracellular traps (NETs) in the pathogenesis of AOSD, and explored the effect of NETs on activating NLRP3 inflammasome and proinflammatory macrophages.

**Methods:**

The sera of 73 AOSD patients and 40 healthy controls were used to detect the level of cell-free DNA and NET-DNA complexes. NET formation ex vivo was analyzed using immunofluorescence and flow plates. The activation of NLRP3 inflammasome in THP-1 cells and proinflammatory macrophages stimulated with DNA purified from NETs was measured using RT-PCR, ELISA, Western blotting and flow cytometry.

**Results:**

The levels of cell-free DNA and NET-DNA complexes were significantly increased in the circulation of patients with AOSD compared with healthy controls, and freshly isolated neutrophils from patients with AOSD were predisposed to high levels of spontaneous NET release. Interestingly, enhanced NET release was abrogated with NADPH oxidase inhibitors and a mitochondrial scavenger. Furthermore, DNA purified from AOSD NETs activated NLRP3 inflammasomes. NET DNA from AOSD also exerted a potent capacity to accelerate the activation of CD68^+^CD86^+^ macrophages and increased the expression of interleukin (IL)-1β, IL-6, and tumor necrosis factor (TNF)-α. Finally, the copy number of mitochondrial DNA (mtDNA) in NETs and plasma was significantly increased in AOSD patients, suggesting that mtDNA may be involved in the activation of NLRP3 and inflammatory macrophages.

**Conclusions:**

These findings implicate accelerated NET formation in AOSD pathogenesis through activation of NLRP3 and proinflammatory macrophages, and identify a novel link between neutrophils and macrophages by NET formation in AOSD.

**Electronic supplementary material:**

The online version of this article (10.1186/s13075-018-1800-z) contains supplementary material, which is available to authorized users.

## Background

Adult-onset Still’s disease (AOSD) is a rare systemic inflammatory disease exhibiting various clinical manifestations typically characterized by a high spiking fever, evanescent skin rash, polyarthralgia, and hepatosplenomegaly [1, 2]. For the etiology, the cytokine storm activated by innate immune cells (mainly neutrophils and monocyte/macrophages) to release proinflammatory molecules, including interleukin (IL)-1β, IL-6, IL-18, tumor necrosis factor (TNF)-α, and macrophage inhibitory factors, plays a crucial role in the pathogenesis of this disease [3, 4]. Unfortunately, little is known about the early events leading to the development of AOSD.

Inflammatory attacks of AOSD are characterized by neutrophilia, suggesting that neutrophils are the primary effector cells in the pathogenesis of AOSD [5, 6]. Neutrophils are the first line of innate immune defense against infection [7]. In addition to phagocytosis, the release of neutrophil extracellular traps (NETs) has been regarded as an effector mechanism of neutrophils in many inflammatory diseases, such as systemic lupus erythematosus (SLE), rheumatoid arthritis (RA), vasculitis, and familial Mediterranean fever (FMF) [8–10]. NETs, the web-like structures released after a novel form of neutrophil activation called NETosis, comprise a chromatin meshwork adhered with granule and nuclear proteins typically present in neutrophil granules, among which are components, such as citrullinated histone 3 (citH3), neutrophil elastase (NE), myeloperoxidase (MPO), etc., with bactericidal activity able to destroy virulence factors [11–13]. However, the role of NETs in AOSD has never been investigated.

Despite the central contributions of neutrophils and monocyte/macrophages to the

pathogenesis of AOSD, the crosstalk between these two types of innate immune cells in AOSD has largely been overlooked. NETs activate NLRP3 (NOD-, LRR- and pyrin domain-containing 3) inflammasome and proinflammatory cytokine production in macrophages in SLE and atherosclerosis [14, 15]. Notably, the increased expression of the NLRP3 inflammasome and elevated serum levels of IL-1β and IL-18 have been reported in patients with AOSD, and these symptoms are positively correlated with disease activity [16, 17]. Of potential relevance to AOSD pathogenesis, we speculated that NETs have the capacity to activate NLRP3 inflammasome and proinflammatory macrophages.

With emerging recognition of the new role of neutrophils as an important component of effectors in immune systems rather than short-lived effectors [18], there is now a compelling reason to explore neutrophil-macrophage interplay. In the present study, we sought to determine whether NET release might be a mechanism by which neutrophil-macrophage interplay predisposes to NLPR3 inflammasome activation in AOSD. We also investigated whether mitochondrial DNA (mtDNA) in AOSD patients predominated the extracellular DNA of NETs.

## Patients and methods

### Subjects

Sera from 73 AOSD patients (51 active and 22 inactive AOSD patients) and 40 healthy controls were included in the present study, and serum samples were collected from all subjects. All patients fulfilled Yamaguchi’s criteria after exclusion of those with infectious, neoplastic, and autoimmune disorders [19]. All serum samples were stored at −80 °C immediately after collection. Information on demographic and clinical data was entered into a database together with the laboratory test results. The AOSD disease activity of each patient was assessed using a modified Pouchot score [20]. The study was performed in accordance with the Declaration of Helsinki and the principles of Good Clinical Practice. Biological samples were obtained under a protocol approved by the Institutional Research Ethics Committee of Ruijin Hospital (ID: 2016–62), Shanghai, China. All subjects signed written informed consent. Table 1 shows the main characteristics of AOSD patients and healthy controls at the time of blood sampling.

**Table 1:** Demographic and clinical characteristics of individuals with adult-onset Still’s disease (AOSD)

Characteristics	Active AOSD (*n* = 51)	Inactive AOSD (*n* = 22)	Healthy controls (*n* = 40)
Age, years	35.8 ± 12.1	37.4 ± 13.9	36.1 ± 10.3
Gender, female/male	40/11	19/3	25/15
Fever	51 (100)	0	0
Evanescent rash	38 (76.4)	0	0
Sore throat	28 (49)	0	0
Arthralgia	37 (70.6)	0	0
Pleuritis	10 (19.6)	0	0
Pneumonia	17 (33.3)	0	0
Pericarditis	4 (7.8)	0	0
Hepatomegaly or elevated liver enzymes	21 (41.2)	0	0
Splenomegaly	15 (29.4)	0	0
Lymphadenopathy	25 (49)	0	0
Myalgia	16 (31.4)	0	0
Abdomen pain	1 (2.1)	0	0
Hemoglobin, g/L	113.5 ± 20.7	127.6 ± 11.4	
Leukocytes, 10^9^/L	17.9 ± 6.2	8.8 ± 2.6	
Platelets, 10^9^/L	301.9 ± 120.1	217.9 ± 115.5	
Ferritin, ng/mL	> 2000	79.6 ± 76.8	
ESR, mm/h	74.0 ± 29.2	12.3 ± 7.7	
CRP, mg/L	87.7 ± 57.3	1.3 ± 0.8	
Systemic score	7 ± 2	0	

All values are presented as number (percentage) or mean ± standard deviation

*CRP* C-reactive protein, *ESR* erythrocyte sedimentation rate

### Quantification of cell-free DNA and NET-DNA complexes in the serum of AOSD patients

Cell-free DNA was quantified in serum using the Quant-iT PicoGreen double-stranded DNA (dsDNA) assay kit (Invitrogen, USA) according to the manufacturer’s instructions. Approximately 10% serum was added per well, followed by incubation for 10 min away from light. NET-DNA complexes, including citH3-DNA, NE-DNA, and MPO-DNA complexes, were quantified using the Quant-iT PicoGreen as previously described [9]. Detailed information is included in Additional file 1.

### Quantitation of NETs

Neutrophils were isolated as previously described [21]. Briefly, heparinized blood from AOSD patients (*n* = 10) and healthy controls (*n* = 10) was isolated by density gradient centrifugation on Polymorphprep (Axis-Shield, Dundee, UK) according to the manufacturer’s instructions. Sytox green (Life Technologies) was used to detect neutrophil extracellular DNA, and PicoGreen (Life Technologies) was used to detect total DNA. Detailed information is included in Additional file 1.

### NET formation monitored by immunofluorescence

We detected NETs using immunofluorescence as previously described [21].

Neutrophils were stimulated with phorbol myristate acetate (PMA; 20 nM; Sigma) for 3.5 h at 37 °C, and fixed with 4% paraformaldehyde. Protein staining was performed using a rabbit polyclonal anti-NE antibody (1:100; Abcam, Serotec, USA) and a mouse monoclonal anti-MPO antibody (1:250; Abcam) overnight at 4 °C. Appropriate fluorochrome-conjugated secondary antibodies (1:100; Jackson ImmunoResearch, West Grove, PA, USA) were applied. The DNA was stained with Hoechst 33342 (Invitrogen). Images were obtained using an Olympus microscope (IX73, Tokyo, Japan). The percentage of NETs was calculated as the average of 5 to 10 fields (× 400) normalized to the total number of neutrophils, and the results are expressed as the mean ± standard deviation (SD).

### Detection of reactive oxygen species (ROS) production

DCFH-DA (Beyotime Institute of Biotechnology, Shanghai, China) and 5 μM MitoSOX (Life Technologies) were used to detect intercellular ROS and mitochondrial superoxide, respectively, according to the manufacturer’s instructions. Flow cytometry and plate reader assays were used for quantitative analysis. Data were analyzed using FlowJo software (Tree Star, Inc., Ashland, OR).

### Quantification of NLRP3 inflammasome activation

THP-1 cells and CD14^+^ monocytes purified from peripheral blood mononuclear cells (PBMCs) by positive magnetic sorting (Miltenyi Biotec, Bergisch Gladbach, Germany) were primed with 100 ng/mL lipopolysaccharide (LPS; Sigma, St. Louis, MO) for 4 h prior to stimulation. Media were subsequently removed and replaced with phenol red-free, serum-free RPMI prior to treatment with 250 ng NET DNA, mtDNA, genomic DNA, RNA, or 5 mM ATP (Sigma, St. Louis, MO) for 2 h. mtDNA, genomic DNA, and RNA were isolated from 293 T cells according to previously published methods [22]. In some experiments, THP-1 cells were incubated or not with the NLRP3 inhibitor MCC950 (10 μM; Invivogen) and DNase I (10 U/mL; Sigma) for 2 h before exposure to NET DNA, and the expression of IL-1β, pro-IL-1β, caspase-1, and NLRP3 in cell lysates (LYS) and IL-1β in supernatants (SN) from THP-1 monocytes was measured by Western blotting. THP-1 SN were concentrated by trichloroacetic acid precipitation [23]. Detailed information is included in Additional file 1.

### Proinflammatory effect of NET-bound DNA in vitro

The macrophage-like state was induced in THP-1 monocytes after exposure to 100 nM PMA for 48 h. Thereafter, the adherent cells were washed with RPMI 1640 medium and incubated for another 24 h to induce a resting state (M0 cells). After stimulating with 250 ng DNA isolated from NETs for 20 h, the mRNA and protein levels of IL-1β, IL-6, and TNF-α were examined using real-time polymerase chain reaction (RT-PCR) and enzyme-linked immunosorbent assay (ELISA). The expression of surface activation markers CD68 and CD86 (BD, USA) was assessed after 24 h using flow cytometry analysis.

### Real-time PCR for mitochondrial copy numbers

mRNA was isolated and cDNA was prepared (PrimeScript RT Master Mix transcription kit, TaKaRa, Tokyo, Japan). RT-PCR was performed to detect mitochondrial and chromosomal genes. The RT-PCR contained 4 ng of DNA, isolated as described above, SYBR green master mix (Takara), and primers for cytochrome B, cytochrome C oxidase subunit III, and GAPDH. The enzyme was activated at 95 °C for 30 s, followed by 40 cycles at 95 °C for 15 s and 60 °C for 60 s. The results are expressed as mtDNA copy numbers [24, 25].

### Statistical analysis

All data were statistically analyzed using SPSS version 20.0 (SPSS Inc., Chicago, IL, USA). Quantitative data are expressed as the means ± SD. Data with a Gaussian distribution was analyzed using an unpaired *t* test or one-way analysis of variance (ANOVA), while nonparametric data were assessed using the Mann-Whitney *U* test or Wilcoxon rank-sum test. *P* values less than 0.05 were considered statistically significant.

## Results

### Elevated sera levels of NETs in the circulation of patients with AOSD

**Fig. 1:**
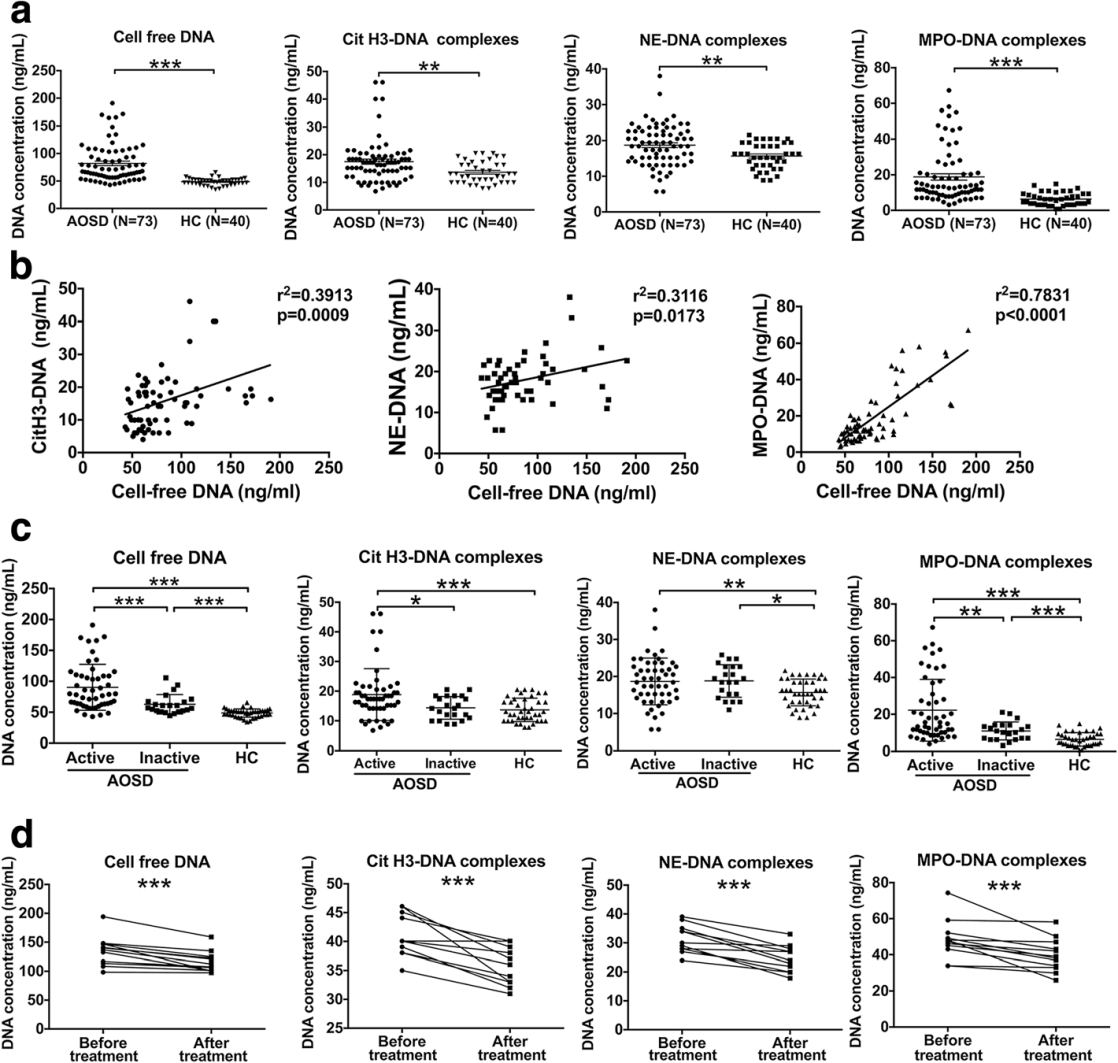
Elevated levels of cell-free DNA and NET-DNA complexes in the circulation of patients with active AOSD. **a** The concentration of cell-free DNA, citrullinated histone 3 (citH3)-DNA, neutrophil elastase (NE)-DNA, and myeloperoxidase (MPO)-DNA complexes in the sera of patients with adult-onset Still’s disease (AOSD; *n* = 73) or healthy controls (HC; *n* = 40) were determined using PicoGreen. **b** Correlation of sera cell-free DNA with citH3-DNA, NE-DNA, and MPO-DNA complexes was analyzed. **c** Levels of sera cell-free DNA, citH3-DNA, NE-DNA, and MPO-DNA complexes in AOSD patients with active disease and inactive disease were measured using PicoGreen. **d** Reduced levels of cell-free DNA, citH3-DNA, NE-DNA, and MPO-DNA complexes in 11 AOSD patients after treatment. The results show the means ± SD. *P < 0.05, **P < 0.01, ***P < 0.001

We first measured the levels of cell-free DNA in the sera of AOSD patients (*n* = 73). Sera analysis displayed a significantly increased level of cell-free DNA in AOSD patients compared with neutrophils from healthy controls, suggesting the potential generation of NETs in vivo (Fig. 1a; *P* < 0.001). To show that circulating cell-free DNA in AOSD patients is primarily derived from NETs, we measured granular components of neutrophils in association with circulating nucleosomes. As citH3, NE, and MPO are prominent constituents of NETs, we assessed the levels of these proteins attached to nucleosomes. Compared with healthy controls, we identified a significantly higher level of citH3-DNA, NE-DNA, and MPO-DNA complexes in sera from AOSD patients (Fig. 1a; *P* < 0.01, *P* < 0.001, and *P* < 0.001, respectively), suggesting that circulating nucleosomes are, at least in part, derived from NET release. Indeed, we found that cell-free DNA showed a significant positive correlation with circulating citH3-DNA, NE-DNA, and MPO-DNA complexes in AOSD patients (Fig. 1b), demonstrating that the cell-free DNA is at least partially neutrophil derived. In addition, the levels of cell-free DNA and NET-DNA complexes were elevated in other autoimmune disease, including SLE and RA (see Additional file 1: Figure S1).

Furthermore, we compared the level of sera NETs in AOSD patients with disease activity. The sera levels of cell-free DNA, citH3-DNA, and MPO-DNA complexes were significantly higher in AOSD patients with active disease (*n* = 51) in relation to those with inactive disease (*n* = 22) (Fig. 1c; *P* < 0.001, *P* < 0.05, and *P* < 0.01, respectively). There was no significant difference in the levels of NE-DNA complexes between AOSD patients with active disease and inactive disease. Moreover, repeated sera from 11 AOSD patients before and after treatments were collected. After treatments, the levels of cell-free DNA and NET-DNA complexes were significantly reduced in patients with AOSD (Fig. 1d; *P* < 0.001). These results showed that circulating NETs were increased during the active phase of AOSD, suggesting the contribution of NETs in the initiation and progression of AOSD.

### Neutrophils from AOSD patients are more prone to release NETs

**Fig. 2:**
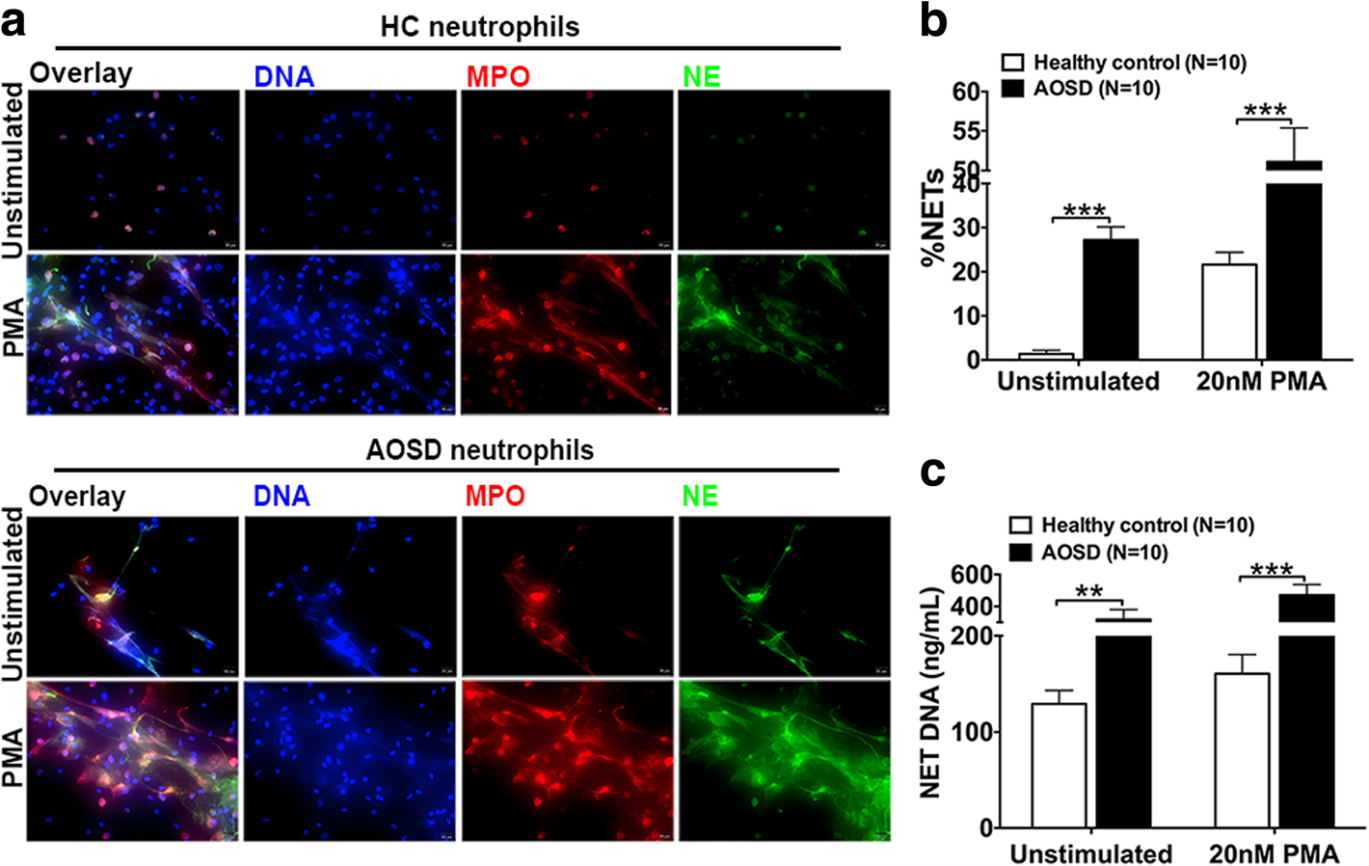
Enhanced NET release in neutrophils from patients with AOSD. **a** Neutrophil extracellular trap (NET) release, determined using immunofluorescence microscopy of neutrophils freshly isolated from healthy controls (HC) or adult-onset Still’s disease (AOSD) patients, seeded onto coverslips and treated with 20 nM phorbol myristate acetate (PMA) for 3.5 h. One representative of ten independent experiments is shown. Blue indicates Hoechst; red indicates myeloperoxidase (MPO); green indicates neutrophil elastase (NE). Original magnification 400×, scale bars = 20 μm. **b** The percentage of NETs (NE, MPO, and Hoechst-labeled neutrophils/total neutrophils) detected in **a** was quantified. **c** NET release from heterologous healthy controls (*n* = 10) or AOSD patients (*n* = 10) for 3.5 h was quantified using PicoGreen. The histograms show the means ± SD. **P < 0.01, ***P < 0.001

To further assess the results of NET release in the circulation of AOSD patients, we isolated peripheral blood neutrophils for the analysis of NET release. Consistent with elevated sera NETs in AOSD patients, neutrophils derived from 10 AOSD patients demonstrated a significantly enhanced propensity to spontaneously form NETs when compared with 10 healthy controls neutrophils (Fig. 2a upper panel and b). Additionally, when stimulated with PMA, a potent NET activator, neutrophils from AOSD patients underwent significantly exaggerated NET formation compared with healthy controls (Fig. 2a lower panel and b). Consistently, the amount of extracellular NET DNA was also increased in AOSD-derived neutrophils, regardless of stimulation (Fig. 2c).

### AOSD neutrophils release NET in a ROS-dependent manner

**Fig. 3:**
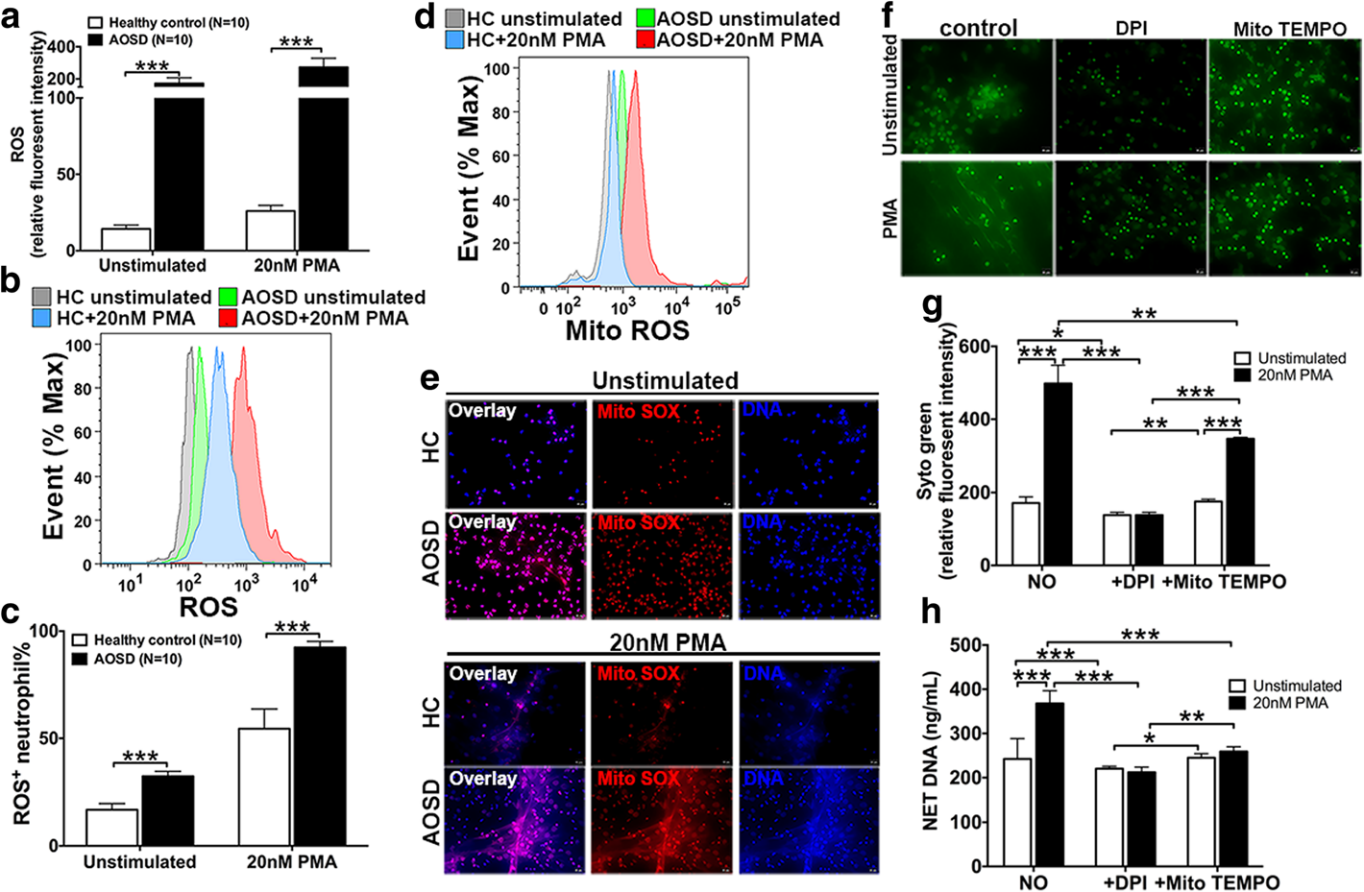
ROS support enhanced NETosis in AOSD patients [39]. Cytoplasmic total reactive oxygen species (ROS) stained with DCFH-DA after the activation of neutrophils with 20 nM phorbol myristate acetate (PMA) for 3.5 h was detected using flow plates (**a**) and flow cytometry (**b** and **c**); the results show the relative fluorescence intensity, ROS-positive neutrophils, and a representative histogram. Mitochondrial ROS production in neutrophils stimulated with 20 nM PMA for 3.5 h and stained with the red fluorescent mitochondrial superoxide indicator, Mito SOX, was determined using flow cytometry (**d**) and immunofluorescence (**e**). Neutrophils from subjects with adult-onset Still’s disease (AOSD; *n* = 10) were stimulated with 20 nM PMA in the presence of the indicated ROS inhibitor. **f**,**g** Sytox green immunofluorescence analysis was performed, and images of neutrophil extracellular trap (NET) formation from each culture were obtained. **h** The quantification of the DNA concentration was achieved using PicoGreen. The figures are representative of six independent experiments. Scale bars = 20 μm. The histograms show the means ± SD. *P < 0.05, **P < 0.01, ***P < 0.001. HC healthy controls

ROS production has been implicated in the induction of NET formation, and NADPH oxidase-mediated pathways and mitochondria are potent sources of ROS [26]. Given that the capacity for NET formation is increased in AOSD patients, we next determined ROS production in neutrophils using intercellular total (DCFH-DA) and the mitochondrial (MitoSOX) ROS indicators. The results showed that intercellular total ROS production was significantly increased in neutrophils from individuals with AOSD compared with healthy controls (Fig. 3a–c). Moreover, mitochondrial superoxide production was also increased in nonstimulated neutrophils from individuals with AOSD compared with healthy controls, as determined using MitoSOX staining (Fig. 3d, e).

To further explore the role of NADPH oxidase and mitochondria in NET formation in AOSD patients, we stimulated neutrophils with PMA in the presence of different ROS inhibitors. DPI, the NADPH oxidase inhibitor, almost completely inhibited PMA-induced NETs (Fig. 3f–h). Superoxide synthesis leading to NETosis has been considered in mitochondrial-dependent pathways [21]. We observed that NET formation was attenuated by MitoTEMPO, the mitochondrial ROS scavenger, suggesting that mitochondrial superoxide production was also necessary for spontaneous NETosis (Fig. 3f–h). Furthermore, we found that DPI had stronger ability to inhibit NET formation than MitoTEMPO (Fig. 3f–h). Taken together, the results indicated that elevated ROS production from both the NADPH oxidase and mitochondrial respiration in AOSD neutrophils is integral for NETosis.

### NET DNA from AOSD activates NLRP3 inflammasomes

**Fig. 4:**
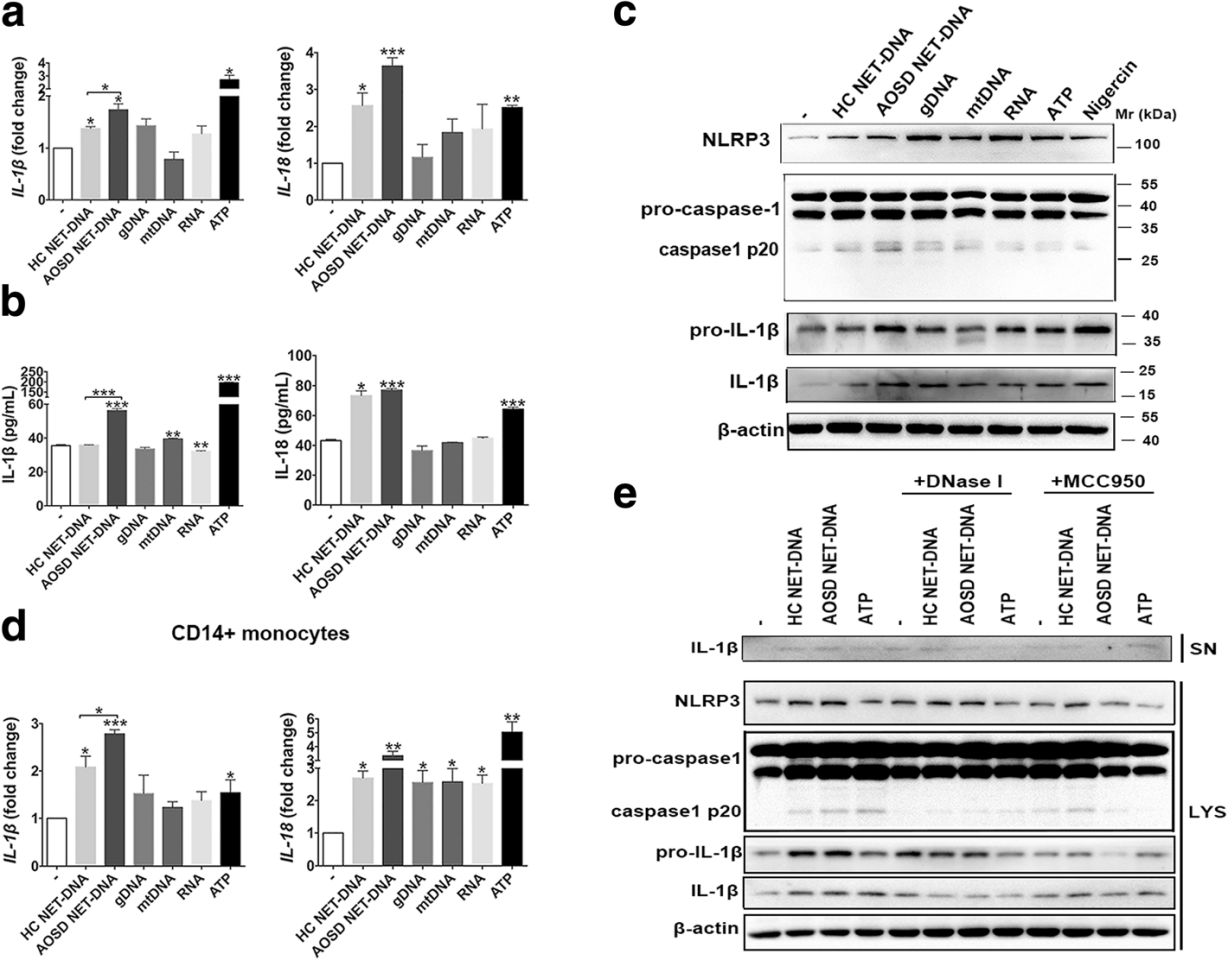
AOSD NETs are potent triggers of NLRP3 inflammasomes. After stimulation with neutrophil extracellular trap (NET) DNA from adult-onset Still’s disease (AOSD) and healthy controls (HC), mitochondrial DNA (mtDNA), genomic DNA g(DNA), and RNA, the expression (**a**) and secretion (**b**) of interleukin (IL)-1β and IL-18 in THP-1 monocytes were measured using RT-PCR and ELISA, respectively. **c** Representative immunoblot analysis for NLRP3 inflammasomes in THP-1 monocytes. The images are representative of three independent Western blot experiments. **d** After stimulation with NET DNA and non-NET source nucleic acids shown above, the expression of IL-1β and IL-18 in PBMC-derived CD14^+^ monocytes from healthy controls was measured using RT-PCR. **e** The expression of IL-1β, pro-IL-1β, and NLRP3 in cell lysates (LYS) and IL-1β in supernatants (SN) from THP-1 monocytes after stimulation with NET-DNA in the presence of the NLRP3 inhibitor MCC950 and DNase I was measured by Western blot. The images are representative of three independent Western blot experiments. The histograms show the means ± SD. *P < 0.05, **P < 0.01, ***P < 0.001

Accumulating evidence has shown that monocytes/macrophages exposed to NETs become proinflammatory and activate the NLRP3 inflammasome. Among the complex components of NETs, extracellular DNA (particularly mtDNA) has important contributions for the proinflammatory properties of NETs. Thus, we subsequently investigated the function of DNA purified from AOSD NETs with regard to the activation of the NLRP3 inflammasome. Priming with LPS, THP-1 monocytes were exposed to DNA from spontaneously formed AOSD NETs or healthy control NETs. mtDNA, genomic DNA, and RNA were used as a control for nucleic acids from a non-NET source. We demonstrated that NET DNA isolated from AOSD patients induced mRNA expression of IL-1β and IL-18, and the levels of IL-1β were significantly higher in THP-1 cells with AOSD NET DNA treatment when compared with NET DNA from healthy controls. The expression levels of IL-18 were upregulated in THP-1 cells with AOSD NET DNA treatment compared with healthy controls; however, this difference did not reach statistical significance (Fig. 4a).

Moreover, ELISA confirmed a more potent ability to secrete IL-1β after exposure to NET DNA from AOSD patients when compared with NET DNA from healthy controls. There was no significant difference in the level of IL-1–8 between AOSD NET DNA and healthy control NET DNA treatments (Fig. 4b).

Caspase-1 and NLRP3 are classical pathways regulating IL-1β and IL-18 expression; thus, we next investigated whether NET DNA can activate caspase-1 and NLRP3. The results showed that both NET DNA from AOSD patients and healthy controls could stimulate the enhanced expression of NLRP3 and caspase-1 at the mRNA level (see Additional file 1: Figure S2). Furthermore, LPS-primed THP-1 cells displayed elevated levels of mature IL-1β, pro-IL-1β, caspase-1, and NLRP3 after stimulation with NET DNA from AOSD, as detected by Western blot (Fig. 4c). We further qualified the results of three independent Western blot experiments by Image J software. We found that AOSD NET DNA induced significantly higher expression of IL-1β compared with healthy control NET-DNA (see Additional file 1: Figure S4). Using PCR and Western blot analysis, the expression levels of IL-1β were upregulated in THP-1 cells with AOSD NET DNA treatment when compared with other DNA stimuli; however, this difference did not reach statistical significance (Fig. 4a, and Additional file 1: Figure S3). Similar results were observed when stimulating CD14^+^ monocytes from healthy controls with AOSD NET DNA at the mRNA (Fig. 4d) and protein levels (see Additional file 1: Figure S4). In the presence of the NLRP3 inhibitor, MCC950, we found that the levels of IL-1β in cell lysates and supernatants were downregulated in THP-1 cells treated with NET-DNA, particularly with AOSD NET DNA (Fig. 4e, and Additional file 1: Figure S5). Furthermore, MCC950 inhibited NLRP3, caspase-1, and pro-IL-1β expression in THP-1 cells with NET DNA treatment (Fig. 4e). Moreover, the addition of DNase I into the monocyte cultures before stimulation with NET DNA inhibited NLRP3 and caspase-1 activation, as well as IL-1β and pro-IL-1β expression (Fig. 4e). Overall, these results indicated that NET DNA from AOSD patients is proinflammatory, exerting its effects by activating NLRP3 inflammasome.

### NET DNA from AOSD activates proinflammatory macrophages

**Fig. 5:**
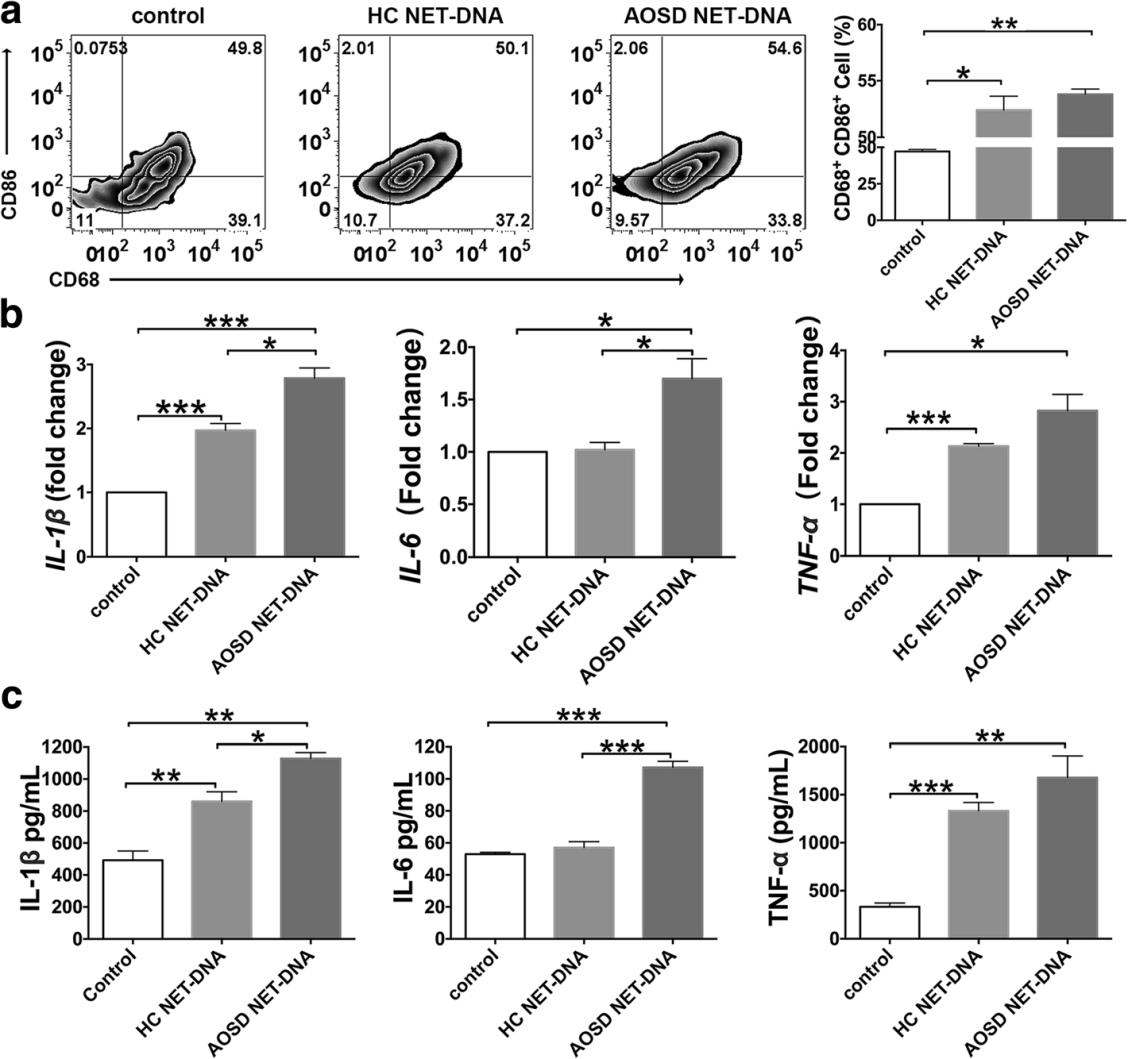
NET DNA activated macrophages are pivotal to the proinflammatory response in AOSD. **a** THP-1-derived macrophages were cultured with purified neutrophil extracellular trap (NET) DNA. After 4 h the cells were stained for cell surface markers using specific monoclonal antibodies or the corresponding isotype controls (right panels). Cells were analyzed using flow cytometry. The percentages of proinflammatory macrophages (CD68^+^CD86^+^) are indicated (left). The levels of mRNA and protein of interleukin (IL)-1β, IL-6, and tumor necrosis factor (TNF)-α in these THP-1-derived macrophages were measured using RT-PCR (**b**) and ELISA analysis (**c**). The histograms show the means ± SD. *P < 0.05, **P < 0.01, ***P < 0.001. AOSD adult-onset Still’s disease, HC healthy controls

Macrophage activation syndrome is a major feature of AOSD, and numerous studies have demonstrated that NETs can prime macrophage activation and proinflammatory cytokine production [27]. Although some NET components such as elastase could affect macrophage function, similar roles for the DNA from NETs have never been explored. Thus, THP-1-derived macrophages were incubated with DNA from AOSD NETs or healthy controls. Using flow cytometry, we observed that CD68^+^CD86^+^, markers of proinflammatory macrophages, were markedly increased in macrophages on stimulation with DNA from AOSD NETs (Fig. 5a). Consistent with the results described above, NET DNA from AOSD neutrophils was more inflammatory, as it potently stimulated both the mRNA and protein expression of IL-1β, IL-6, and TNF-α in THP-1-derived macrophages (Fig. 5b, c). Macrophages with NET DNA from healthy controls tended to have lower expression levels of IL-1β, IL-6, TNF-α, and CD68^+^CD86^+^ than AOSD NET DNA-treated macrophages, although there were no significant differences in the levels of TNF-α and CD68^+^CD86^+^ (Fig. 5a–c). Considering enhanced spontaneous NETs in patients with AOSD, it can be shown that NET DNA from AOSD patients has a more enhanced capacity to facilitate proinflammatory macrophages in vivo. Overall, these results suggest that the circulating DNA present in AOSD patients (as shown in Fig. 1a) could activate NLRP3 inflammasomes and proinflammatory macrophages.

### NET-derived mtDNA is increased in AOSD patients

**Fig. 6:**
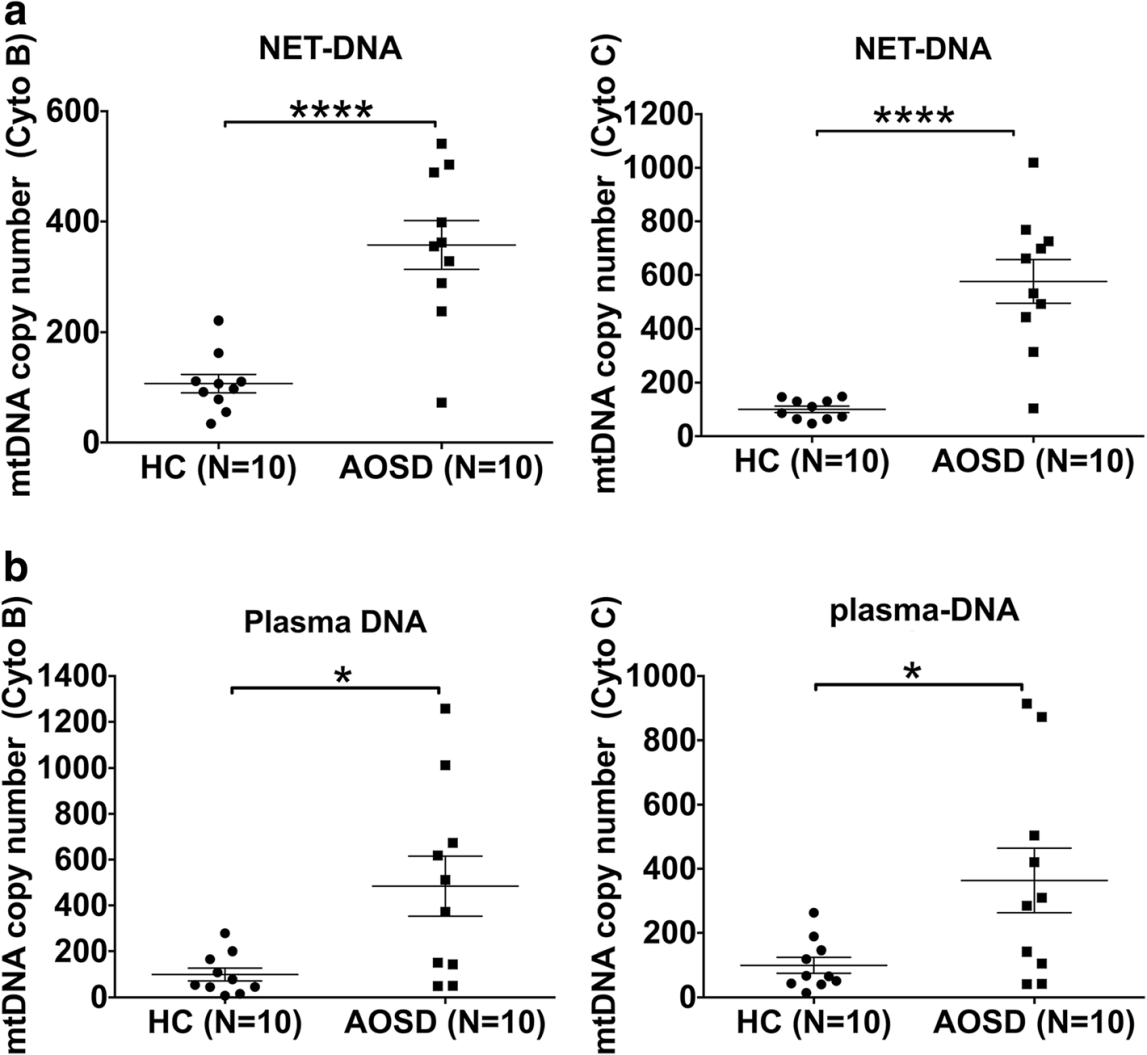
Enriched mitochondrial DNA in AOSD NETs and plasma. Mitochondrial copy numbers from neutrophil extracellular traps (NETs) (**a**) and plasma (**b**) were determined using RT-PCR. Symbols represent individual samples; horizontal and vertical lines show the means ± SD. *P < 0.05, ****P < 0.0001. AOSD adult-onset Still’s disease, HC healthy controls, mtDNA mitochondrial DNA

mtDNA exerts proinflammatory effects on monocytes/macrophages [28, 29]. To confirm our hypothesis that the function of NET DNA might, to a large extent, rely on the high levels of mtDNA in AOSD, we assessed the levels of mtDNA in NETs and plasma from AOSD patients. DNA spontaneously released from AOSD NETs was highly enriched for mtDNA compared with NETs from healthy control neutrophils (Fig. 6a). Consistently, the mtDNA copy number was significantly higher in the plasma of individuals with AOSD than that in healthy controls (Fig. 6b). Overall, these observations suggest that mtDNA present in NETs can significantly activate the macrophage phenotype and endow these cells with proinflammatory capabilities.

## Discussion

In the present study, we describe a novel role for neutrophils in the induction and regulation of inflammation in AOSD through NET release. Herein, we provide the first evidence that mtDNA-bearing NETs are released from neutrophils in patients with AOSD, contributing to the activation of NLRP3 inflammasomes, and stimulating macrophages for proinflammatory cytokine production.

The activation of neutrophils and macrophages is a hallmark of AOSD; however, the link between these features in AOSD remains unclear. The present study is the first to elucidate the crosstalk between these two cells. Neutrophils act as the first line of immune defense to migrate to the site of inflammation, where these cells eliminate pathogens via phagocytosis and cytokine production [18, 30]. Unlike apoptosis and necrosis, increasing numbers of studies have paid close attention to a distinct process of neutrophil death called NETosis, placing this process at the center of various pathological states [26, 31]. While various groups have suggested the participation of these structures in the development of autoinflammatory diseases, such as FMF and IL-1β-dependent disorders [32], their role and pathogenic mechanism in AOSD remain unclear. The AOSD proinflammatory milieu, characterized by increased IL-1β, IL-6, and IL-18, is highly conducive for the induction of NETosis. Therefore, this phenomenon perpetuates a vicious cycle, leading to the inflammatory cascade of a NET-driven positive feedback mechanism.

Nucleic acids constitute a major molecular pattern recognized during infections with viruses and intracellular bacteria. However, chronic or inappropriate inflammatory signaling mediated by nucleic acid has been implicated in inflammatory and autoimmune diseases [33]. NETs provide the immune system with access to enhanced sources of extracellular DNA, particularly mtDNA. mtDNA is a critical pathogenic component of NETs. Unlike genomic DNA, mtDNA contains hypomethylated CpG motifs similar to bacterial DNA and has recently been reported as a potent agonist of the innate immune system [23, 34]. Circulating mtDNA in response to cellular damage and stress stimuli has been implicated in the inflammatory pathology of diverse diseases, including RA, atherosclerosis, and heart disease [34–36]. Studies have revealed that mtDNA contributes to NLRP3 inflammasome activation [37]. In the present study, we identified two novel roles for mitochondria in AOSD: first, the capability of mitochondrial ROS to promote NETosis; second, the release of mtDNA with potent proinflammatory properties.

The NLRP3 inflammasome is a key inducer of inflammation in response to pathogens and innate immune stimuli. The NLRP3 inflammasome is clearly involved in the pathogenesis of diabetes, atherosclerosis, and infectious disease due to the secretion of IL-1β and IL-18 from this complex [38]. Recent evidence suggests that aberrant activation of NLRP3 inflammasome in AOSD is positively correlated with disease activity [17], whereas the molecular mechanisms implicated in NLRP3 regulation remain incompletely characterized in AOSD. Clear evidence supports mtDNA as an endogenous agonist of inflammasomes by activating Toll-like receptor (TLR)9 [28, 37]. Moreover, circulating mtDNA has been implicated in the TLR9-dependent inflammatory pathology of diverse diseases such as RA, atherosclerosis, and acute liver injury [29], and we hypothesized that AOSD neutrophils spontaneously release NETs enriched in mtDNA, leading to enhanced proinflammatory potential mainly via TLR9, while many mechanistic questions will need to be determined in our future studies.

NADPH oxidase-dependent ROS generation is necessary for NETosis in most contexts; however, NADPH oxidase is not the sole source of neutrophil ROS. Indeed, mitochondria are one of the major sites of ROS generation, and enhanced mitochondrial ROS has been associated with chronic inflammatory conditions. In this study, we have shown that enhanced NET formation in AOSD is dependent on both NADPH oxidase and mitochondrial-derived ROS.

Beyond nucleic acids, it is likely that other molecules present in NETs, including proteins and oxidized mtDNA, could trigger other inflammasome machinery in addition to NLRP3. Future studies should focus on identifying other NET components that exert proinflammatory functions.

## Conclusion

In our study, we demonstrated that the onset of the AOSD crisis is characterized by potent NET formation, leading to activation of NLRP3 inflammasome and proinflammatory macrophages. These findings, explain, in part, the proinflammatory cascade in AOSD that potentially characterizes various neutrophil-associated disorders. Furthermore, NET formation provides a link between neutrophils and macrophages to enhance a cytokine storm.

## Additional file

Additional file 1: Supplementary methods and Figures S1–S5. (DOCX 2226 kb)

## Abbreviations

AOSD: Adult-onset Still’s disease
citH3: Citrullinated histone 3
FMF: Familial Mediterranean fever
MPO: Myeloperoxidase
mtDNA: Mitochondrial DNA
NE: Neutrophil elastase
NET: Neutrophil extracellular trap
RA: Rheumatoid arthritis
ROS: Reactive oxygen species
SLE: Systemic lupus erythematous
