# Supplementary material for: Increased neutrophil extracellular traps activate NLRP3 and inflammatory macrophages in adult-onset Still’s disease
Source: Arthritis Res Ther. 2019 Jan 7;21:9. doi: 10.1186/s13075-018-1800-z (PMC6323819; doi:10.1186/s13075-018-1800-z)
Supplement: Supplementary file 1 — Supplementary methods and Figures S1–S5. (DOCX 2226 kb) [file 13075_2018_1800_MOESM1_ESM.docx]

**Increased Neutrophil Extracellular Traps Activate NLRP3 and Inflammatory Macrophages in Adult-onset Still’s Disease**

Qiongyi Hu *, Hui Shi *, Ting Zeng, Honglei Liu, Yutong Su, Xiaobing Cheng, Junna Ye, Yufeng Yin, Mengru Liu, Hui Zheng, Xinyao Wu, Huihui Chi, Zhuochao Zhou, Jinchao Jia, Yue Sun ^#^, Jialin Teng ^#^, Chengde Yang ^#^

Department of Rheumatology and Immunology, Ruijin Hospital, Shanghai Jiao Tong University School of Medicine, No. 197 Ruijin Second Road, Shanghai, 200025, China.

* These authors contributed equally to this work.

**^#^Corresponding authors:**

Chengde Yang, Department of Rheumatology and Immunology, Ruijin Hospital, Shanghai Jiao Tong University School of Medicine, Shanghai 200025, China

Tel.: (86)-21-64370045ext665130; Fax: (86)-21-54109718

Email: yangchengde@sina.com; or to Jialin Teng,

Email: tengteng8151@sina.com; or to Yue Sun, Email: winniesun2015@163.com

**Supplementary Methods**

**Quantification of cell-free DNA and NETs-DNA complexes in the serum of AOSD patients**

Cell-free DNA was quantified in serum using the Quant-iT PicoGreen double-stranded DNA (dsDNA) assay kit (Invitrogen, USA) according to the manufacturer’s instructions. Approximately 10% serum was added per well, followed by incubation for 10 min away from light. NE-DNA, MPO-DNA and citH3-DNA complexes were quantified using the Quant-iT PicoGreen as previously described (12). As the capturing antibody, anti-citH3, NE and MPO monoclonal antibody (Abcam, Serotec, USA) was coated onto 96-well microtiter plates (75 μl per well) overnight at 4°C. After blocking in 1% BSA (125 μl per well) for 90 min at room temperature, 10% serum was added per well, followed by incubation overnight at 4°C. The plate was washed 5 times, followed by the addition of Pico Green from the kit described above.

**Isolation of neutrophils from human peripheral blood**

Neutrophils were isolated as previously described (25). Briefly, heparinized blood from AOSD patients (N=10) and healthy controls (N=10) was isolated by density gradient centrifugation on Polymorphprep (Axis-Shield, Dundee, UK) according to the manufacturer’s instructions. Erythrocytes were lysed in a hypertonic solution, and neutrophils were resuspended in RPMI 1640 (Life Technologies, Waltham, MA) containing 0.01 M HEPES (Gibco, Gaithersburg, MD), MEM non-essential amino acids (Thermo Scientific, Waltham, MD) and 10% fetal calf serum (FCS).

**Quantification of NET-DNA**

Neutrophils (1 × 10^6^ cells/ml) were seeded in 96-well plates in the presence or absence of the indicated inhibitors (all obtained from Sigma, St. Louis, MO), DPI (25 µM) and MitoTEMPO (10 µM), for 1 h and subsequently activated with PMA (20 nM) for 3.5 h. NETs were detached for 10 min at 37°C using diluted nuclease buffer. The nuclease activity was stopped using 5 mM EDTA, and the culture supernatants were collected and stored at 4℃ until further use. Sytox green (167 nM, Life Technologies) was used to detect extracellular DNA, and Pico Green (1:500) was used to detect total DNA. Fluorescence was quantified using the BIOTEK Synergy HTX plate reader and visualized using immunofluorescence microscopy; the only demonstrable staining after this short incubation period was that of extracellular DNA, and no intact nuclear DNA could be visualized. Representative images were captured using an Olympus microscope (IX73).

**Isolation of NET DNA**

To prepare DNA derived from NETs as a stimulant, neutrophils from AOSD patients and healthy controls (1x10^6^ cells/well) were seeded in 6-well plates for 30 min in a total volume of 1 ml RPMI-1640 medium and subsequently treated with 20 nM PMA for 3.5 h to induce NETs. Subsequently, the cells were washed twice with fresh RPMI-1640 medium, and the NETs were collected by extensively pipetting with 1 ml RPMI-1640 medium. Thereafter, the NETs were recovered as a supernatant at a concentration of 1x10^6^ cell equivalents/ml using centrifugation at 400 x g and 10,000 x g and stored at -80°C. DNA from supernatants of PMA-stimulated neutrophils was purified using the QIAamp DNA Mini kit (Qiagen, USA), and the concentration of the purified DNA was qualified using a Nano-drop (Thermo Scientific, Waltham, MA).

**Quantification of NLRP3 inflammasome activation**

CD14^+^ monocytes purified from PBMC by positive magnetic sorting (Miltenyi Biotec, Bergisch Gladbach, Germany) and THP-1 cells were primed with 100 ng/ml of LPS (Sigma) for 4 h prior to stimulation. Media was subsequently removed and replaced with phenol-red-free, serum-free RPMI prior for treatment with 250 ng NETs DNA, mitochondrial DNA, genomic DNA, RNA or 5 mM ATP (Sigma) for 2 h. Following treatment, the media was collected for the quantification of IL-1β using cytometric bead-array assay (BD, USA) and IL-18 using ELISA (R&D, Minneapolis, USA) according to manufacturer’s instructions, and the gene expression of IL-1β and IL-18 was analyzed using RT-PCR. The expression of NLRP3, active caspase-1, pro- IL-1β and IL-1β was examined using Western blotting.

**Western blotting**

To evaluate active caspase-1 using Western blotting, the cells were stimulated as described above and subsequently lysed in 1x SDS sample buffer. The protein concentration in the cell lysate was detected using the BCA Protein Assay kit (Beyotime Institute of Biotechnology, Shanghai, China). Equal quantities of protein (20 μg) were separated using 10% SDS-PAGE and transferred to a PVDF membrane (Millipore, USA). After blocking with 5% non-fat milk in TBST (TBS with 0.1% Tween-20, pH 7.4) at room temperature for 2 h, the membranes were incubated at 4°C overnight with the following primary antibodies: rabbit monoclonal anti-NLRP3 (1:1000, CST, USA), rabbit polyclonal anti-CASP1 (1:1000, CST, USA), goat polyclonal anti-pro-IL-1β (1:2000, R&D) and rabbit polyclonal anti-IL-1β (1:1000, BioVision, USA). The membranes were washed three times and incubated with HRP-conjugated secondary antibody (CST) at room temperature for 1 h. Anti-beta-actin antibody was used as an internal control. Peroxidase was visualized using an enhanced chemiluminescence system (ECL) (Millipore). Bands were quantitated using Image J 1.48 software (Bio-Rad, USA), and results are expressed as fold change relative to the internal control.

**Supplementary Figures**

**
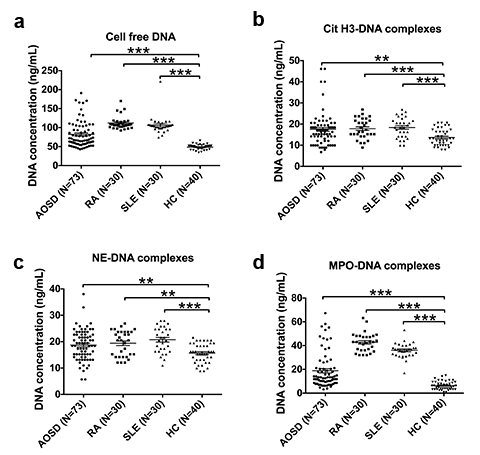
**

**Figure S1.** The concentration of cell-free DNA(a), citH3-DNA (b), NE-DNA (c) and MPO-DNA complexes (d) in the sera of patients with AOSD (N=73), RA (N=30), SLE (N=30) and healthy controls (N=40) were determined using PicoGreen. **=P < 0.01, ***=P < 0.001.

**
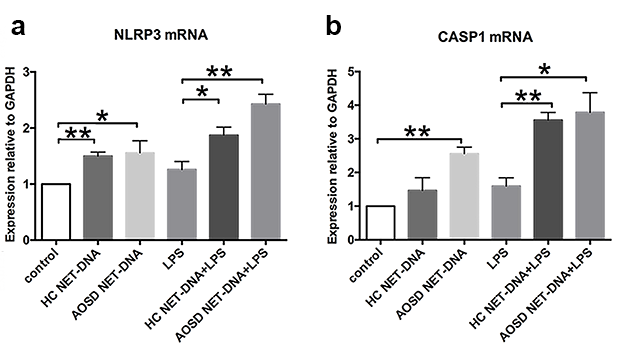
**

**Figure S2.** With NET DNA from AOSD and healthy control stimulation, NLRP3 and caspase-1 mRNA levels in THP-1 cells were determined using RT-PCR. *=P <0.05, **=P < 0.01.


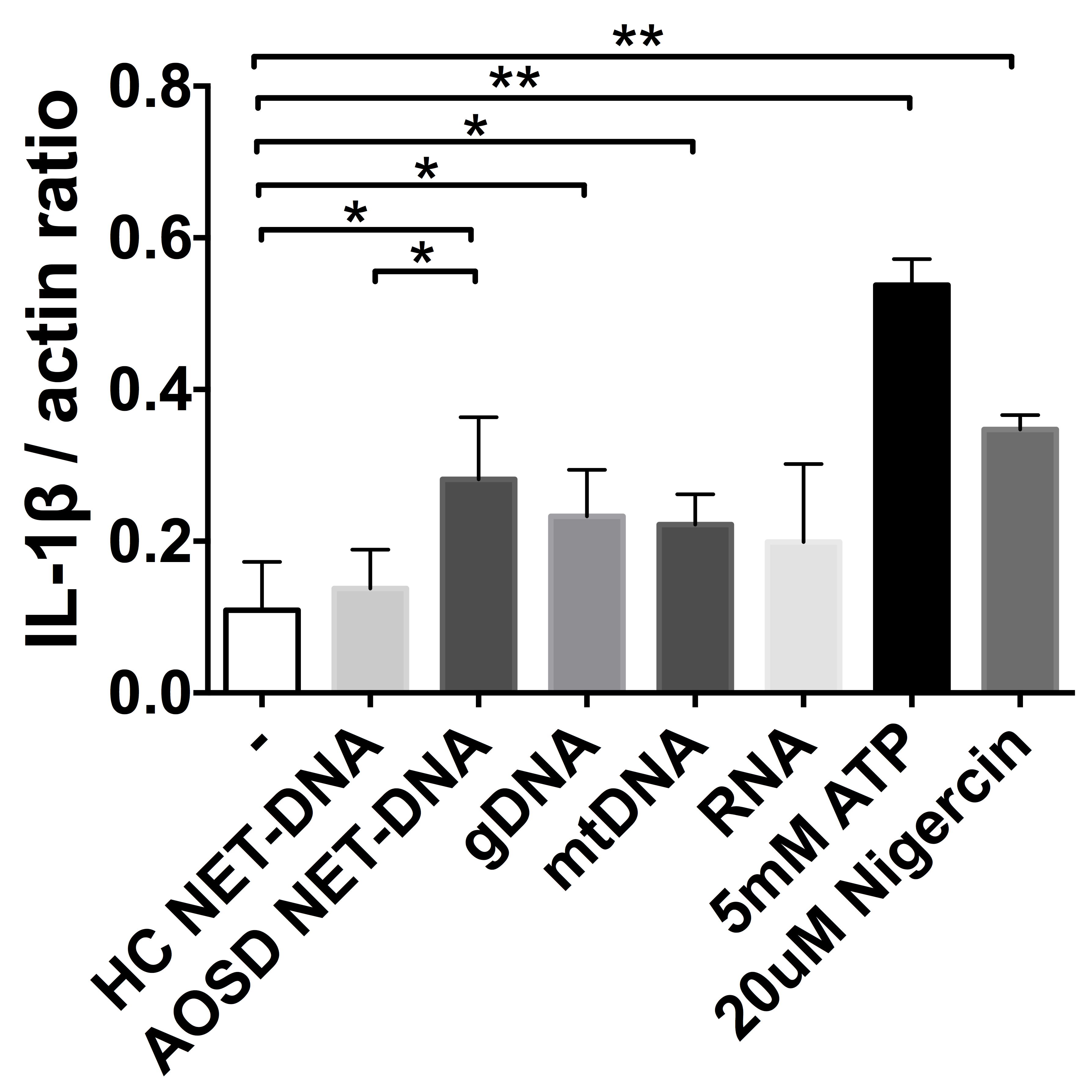


**Figure S3.** With NET DNA from AOSD and healthy control stimulation, the expression levels of IL-1β detected by WB were qualified by Image J software. *=P <0.05, **=P < 0.01.

**
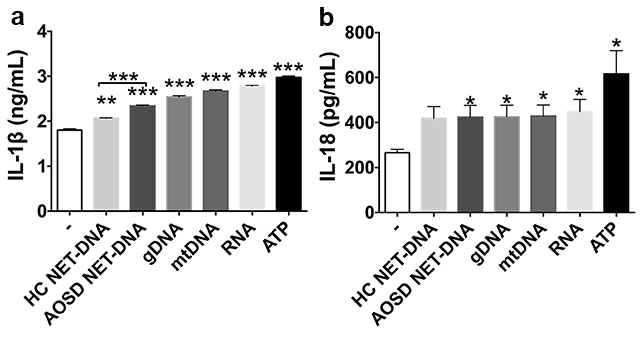
**

**Figure S4.** After stimulation with NET DNA and non-NET source nucleic acids, the expression of IL-1β and IL-18 in CD14^+^ monocytes from healthy controls were measured using ELISA. *=P <0.05, **=P < 0.01.


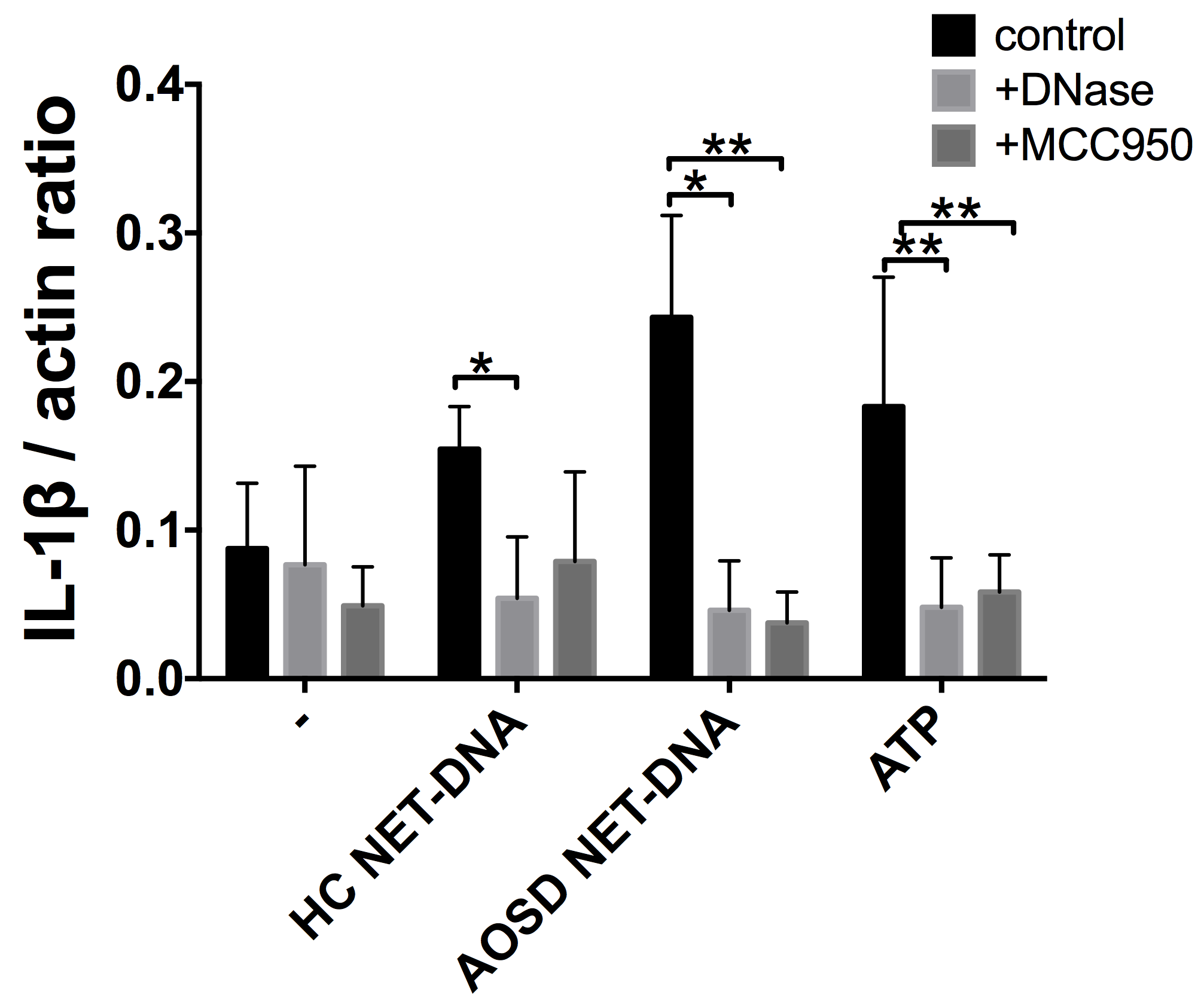


**Figure S5.** In the presence of MCC950 and DNase, the expression levels of IL-1β in THP-1 cells with or not NET-DNA treatment detected by WB were qualified by Image J sofeware. *=P <0.05, **=P < 0.01.
